# Blocking GM-CSF receptor α with mavrilimumab reduces infiltrating cells, pro-inflammatory markers and neoangiogenesis in ex vivo cultured arteries from patients with giant cell arteritis

**DOI:** 10.1136/annrheumdis-2021-220873

**Published:** 2022-01-19

**Authors:** Marc Corbera-Bellalta, Roser Alba-Rovira, Sujatha Muralidharan, Georgina Espígol-Frigolé, Roberto Ríos-Garcés, Javier Marco-Hernández, Amanda Denuc, Farah Kamberovic, Patricia Pérez-Galán, Alexandra Joseph, Annalisa D’Andrea, Kent Bondensgaard, Maria C Cid, John F Paolini

**Affiliations:** 1 Vasculitis Research Group, Department of Autoimmune Diseases, Hospital Clínic, University of Barcelona, Institut d’Investigacions Biomèdiques August Pi i Sunyer (IDIBAPS), Barcelona, Spain; 2 Kiniksa Pharmaceuticals Corp, Lexington, Massachusetts, USA; 3 Biobank, IDIBAPS, Barcelona, Spain; 4 Department of Hematology-Oncology, IDIBAPS, Barcelona, Spain

**Keywords:** cytokines, chemokines, systemic vasculitis

## Abstract

**Background:**

Effective and safe therapies are needed for the treatment of patients with giant cell arteritis (GCA). Emerging as a key cytokine in inflammation, granulocyte-macrophage colony stimulating factor (GM-CSF) may play a role in promoting inflammation in GCA.

**Objectives:**

To investigate expression of GM-CSF and its receptor in arterial lesions from patients with GCA. To analyse activation of GM-CSF receptor-associated signalling pathways and expression of target genes. To evaluate the effects of blocking GM-CSF receptor α with mavrilimumab in ex vivo cultured arteries from patients with GCA.

**Methods:**

Quantitative real time PCR, in situ RNA hybridisation, immunohistochemistry, immunofluorescence and confocal microscopy, immunoassay, western blot and ex vivo temporal artery culture.

**Results:**

GM-CSF and GM-CSF receptor α mRNA and protein were increased in GCA lesions; enhanced JAK2/STAT5A expression/phosphorylation as well as increased expression of target genes CD83 and Spi1/PU.1 were observed. Treatment of ex vivo cultured GCA arteries with mavrilimumab resulted in decreased transcripts of CD3ε, CD20, CD14 and CD16 cell markers, and reduction of infiltrating CD16 and CD3ε cells was observed by immunofluorescence. Mavrilimumab reduced expression of molecules relevant to T cell activation (human leukocyte antigen-DR [HLA-DR]) and Th1 differentiation (interferon-γ), the pro-inflammatory cytokines: interleukin 6 (IL-6), tumour necrosis factor α (TNFα) and IL-1β, as well as molecules related to vascular injury (matrix metalloprotease 9, lipid peroxidation products and inducible nitric oxide synthase [iNOS]). Mavrilimumab reduced CD34 + cells and neoangiogenesis in GCA lesions.

**Conclusion:**

The inhibitory effects of mavrilimumab on multiple steps in the GCA pathogenesis cascade in vitro are consistent with the clinical observation of reduced GCA flares in a phase 2 trial and support its development as a therapeutic option for patients with GCA.

**Key messages**

**What is already known about this subject?**

- GM-CSF transcripts were detected in temporal arteries from patients with giant cell arteritis (GCA) more than two decades ago.
- More recently, GM-CSF protein has been shown to be produced and secreted by peripheral blood mononuclear cells from patients with active GCA and detected in GCA-involved temporal arteries by immunohistochemistry.
- Expression of GM-CSF receptor and its functional role in GCA lesions has not been previously explored.

**What does this study add?**

- The study demonstrates expression of GM-CSF and its receptor in distinct cell subsets in GCA lesions.
- Moreover, GM-CSF receptor signalling is activated, and expression of typical target genes is increased.
- Exposure of ex-vivo cultured arteries to mavrilimumab reduces CD16 and CD3ε cell infiltration and reduces key molecules involved in T cell activation and differentiation, expression of pro-inflammatory cytokines, markers of vascular injury and neoangiogenesis.
- Taken together, these data point towards a relevant role of GM-CSF in the development of vascular inflammation and injury in GCA.

**How might this impact on clinical practice or future developments?**

- The clear impact of mavrilimumab on key steps in the pathogenesis of GCA supports its further development as a therapeutic option for patients with GCA.

## Introduction

Giant cell arteritis (GCA) is a chronic inflammatory condition affecting large and medium arteries in individuals older than 50 years. Common manifestations include headache, scalp tenderness, polymyalgia rheumatica and systemic symptoms.^1 2^ Inflammation-induced vascular remodelling results in ischaemic complications or aneurysms.^3^

High-dose glucocorticoids (GCs) dramatically improve symptoms of GCA, but relapses occur in 34%–75% of patients when GCs are tapered,^4–6^ leading to prolonged treatment and frequent GC-associated side effects.^7 8^ Blocking the interleukin 6 (IL-6) receptor with tocilizumab (TCZ) demonstrated efficacy in reducing relapses, sparing GC,^9 10^ and improving quality of life.^11^ However, more than 40% of patients treated with TCZ are unable to maintain GC-free remission and about 60% of responders relapse on discontinuation,^12^ indicating heterogeneity in response and underlining the need for alternative therapeutic options. TCZ also inhibits synthesis of acute-phase reactants, even without full suppression of disease activity, rendering their use unreliable for monitoring of disease flare.^13 14^

The search for additional therapeutic targets in GCA is hampered by the limited understanding of pathogenesis. Studies indicate that genetics, ageing and immune responses against unknown antigen(s) likely play a major role.^15 16^ Dendritic cells activated by innate immune mechanisms may drive adaptive immunity by stimulating T lymphocytes and promoting their differentiation into Th1 and Th17 effector cells.^17–24^ Concomitant and subsequent activation of macrophages amplifies inflammatory loops, leading to vascular injury and remodelling.^25–27^

GM-CSF is a pro-inflammatory cytokine produced by fibroblasts, epithelial, endothelial, myeloid and T cells on stimulation with other cytokines or pathogen-associated molecular pattern molecules.^28–30^ GM-CSF has a seminal role in disease progression in animal models of inflammatory conditions.^28–30^ GM-CSF receptor is composed of an alpha-chain conferring specificity and a signalling beta-chain shared with other cytokine receptors (IL-3, IL-5 and IL-34).^28–30^ On GM-CSF binding, the receptor beta-chain predominantly signals through JAK2–STAT5 pathway. GM-CSF acts primarily on myeloid cells, promoting activation of dendritic cells and macrophages and differentiation of monocytes into dendritic cells, but other cell types may also respond.^28–30^ GM-CSF mRNA has been detected in arterial lesions of GCA, and GM-CSF protein production by circulating peripheral blood mononuclear cells from GCA patients is increased compared with healthy controls.^22 24^ According to its known biological functions, GM-CSF may have a role in promoting and amplifying vascular inflammation and injury in GCA.

Mavrilimumab is a fully human IgG4 monoclonal antibody able to neutralise GM-CSF effects by binding to the GM-CSF receptor alpha chain (GM-CSFRα).^31^ In a phase 2b trial in patients with rheumatoid arthritis, mavrilimumab showed comparable efficacy to anti-TNFα blocker golimumab and superior efficacy compared with placebo, as well as a good safety profile.^32–34^ The putative role of GM-CSF in critical steps of GCA pathogenesis suggests therapeutic potential for mavrilimumab in this disease, supported by a recent phase 2 trial.^35^

This study aimed to investigate the expression of GM-CSF and GM-CSFRα in inflamed arteries from patients with GCA, to detect activation of GM-CSFR-related signalling pathways and modulation of downstream gene expression, and to investigate the impact of GM-CSFRα blockade with mavrilimumab on inflammation in ex vivo cultured arteries from patients with GCA.

## Patients and methods

### Patients

The study investigated samples from four different patient groups according to the processing of their biospecimens (clinical characteristics of patients, controls and their samples: online supplemental table S1).

10.1136/annrheumdis-2021-220873.supp1 Supplementary data

### Temporal artery culture

Details have been previously described^36^ and are available in online supplemental methods.

### In situ RNA hybridisation

RNAScope (RS) (ACDbio, Abingdon, UK) in situ hybridisation was performed on formalin-fixed paraffin-embedded (FFPE) sections of GCA and control temporal artery biopsies to detect transcripts of specific genes, including GM-CSF, GM-CSFRα, CD83 and Spi1 (PU.1). After fixation and sectioning, tissue was permeabilised and probed with target-specific double Z probes specific to single target mRNA, and hybridisation signals were further amplified for detection. Visualised with a microscope, each red dot represents a single target mRNA molecule. Expression score was calculated as RS score (dots/cell) multiplied by positivity score (% cells positive with >1 dot/cell) (online supplemental table S2).

### Candidate gene expression analysis

Candidate genes relevant to the immunopathogenesis of GCA were selected according to the current pathogenesis model^15 16^ and known effects of GM-CSF in experimental systems.^28 29^ Transcripts were detected by quantitative real-time PCR, details of RNA extraction, reverse transcription and fluorescence quantification are provided in the online supplemental methods (online supplemental table S3).

### Immunohistochemistry

Two micrometre thick temporal artery sections from FFPE samples were used for immunohistochemistry. After 20-minute antigen retrieval with citrate buffer (pH 6), samples were immunostained with specific antibodies, using the Leica Microsystems’ Bond-max automated immunostainer and the Bond Polymer Refine Detection System (Leica Microsystems), developed with diaminobenzidine and counterstained with haematoxylin (antibodies used, dilutions and optimised incubation times: online supplemental table S4-C). Positive and negative control tissues for protocol optimisation were selected from Human Protein Atlas (www.proteinatlas.org) and obtained from Institut d'Investigacions Biomèdiques August Pi i Sunyer Biobank.

### Immunofluorescence

Immunofluorescence staining and imaging were performed with fresh-frozen or cultured temporal artery sections (online supplemental methods and online supplemental table S4-A).

### Protein detection by western blot

Fresh-frozen temporal artery biopsies (TABs) from three patients with GCA and three controls were processed as described in online supplemental table S4-B.

### Detection of proteins in the supernatants of cultured arteries and patient sera

Cytokines, chemokines or membrane-bound molecules released into artery culture supernatants were detected by immunoassay (online supplemental table S5).

### Statistical analysis

Non-parametric Mann-Whitney U test and Wilcoxon matched-pairs signed rank test were used for unpaired and paired data analysis, respectively, using Graphpad Prism software.

## Results

### GM-CSF and GM-CSFRα expression is increased in GCA lesions

**Figure 1:**
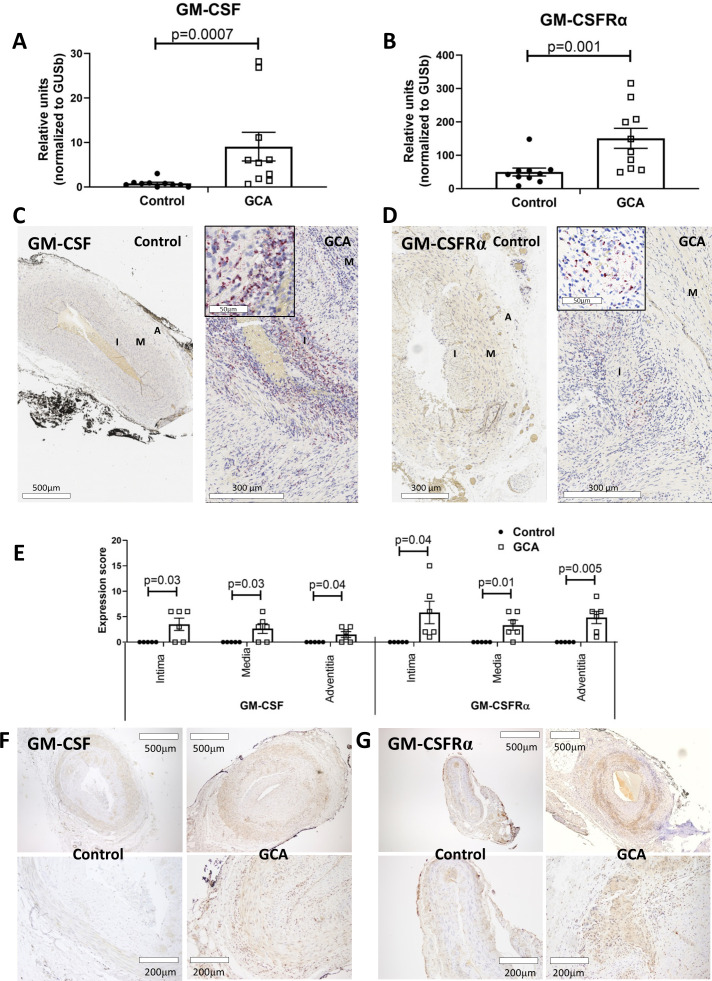
Granulocyte-macrophage colony stimulating factor (GM-CSF) and GM-CSFRα expression in GCA lesions. Concentrations of GM-CSF (A) and GM-CSFRα mRNA (B) measured by qRT-PCR in fresh-frozen histologically negative arteries (controls) (n=10) vs GCA-positive arteries (n=10). Results are expressed in relative units normalised to the housekeeping transcript *GUSB*. GM-CSF (C) and GM-CSFRα (D) RNA hybridisation signals (red dots) on control temporal arteries and GCA-involved arteries. (E) Quantitation of RS signal (expression score) in different arterial layers in 6 GCA-involved and 5 control arteries. Immunostaining with anti-GM-CSF (F) and anti-GM-CSFRα (G) antibodies (brown colour) of FFPE normal or GCA-involved arteries (representative of 5 controls and 12 GCA arteries). A, adventitia layer; FFPE, formalin-fixed paraffin-embedded; GCA, giant cell arteritis; GM-CSFRα, GM-CSF receptor alpha chain; I, intima layer; M, media layer; qRT-PCR, quantitative real-time PCR; RS, RNAScope.

GM-CSF and GM-CSFRα transcripts were increased in homogenised temporal artery biopsies from patients with GCA, whereas GM-CSF mRNA was virtually undetectable, and GM-CSFRα expression was very low in control arteries (figure 1A, B). Transcripts for GM-CSF or GM-CSFRα mRNA were clearly detectable by in situ RNA hybridisation in all arterial layers of GCA biopsies, whereas virtually no signal for either gene product was detectable in control arteries (figure 1C–E).

Immunostaining confirmed the presence of GM-CSF and GM-CSFRα protein on infiltrating inflammatory cells and endothelial cells in GCA arteries. In contrast, no GM-CSF protein and only low levels of GM-CSFRα protein were detected in control arteries (figure 1F, G).

**Figure 2:**
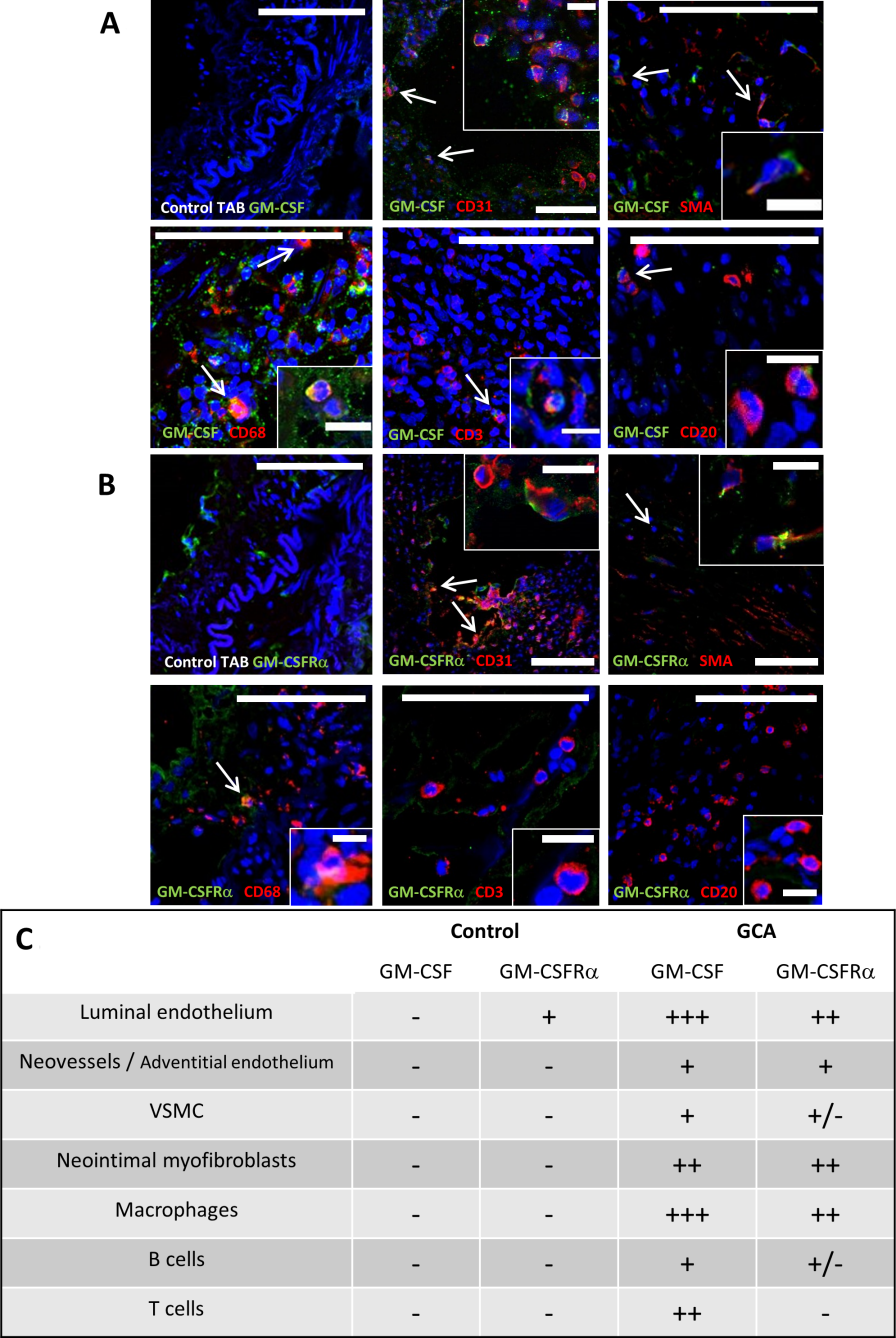
GM-CSF and GM-CSFRα expression by immune and resident cells. Merged double immunofluorescence staining with anti-GM-CSF (A) or anti-GM-CSFRα (B) antibodies (both in green) and cell surface markers CD68 (macrophages), CD31 (endothelial cells), CD3 (T lymphocytes), CD20 (B lymphocytes) and SMA (identifying vascular smooth muscle cells and myofibroblasts) (all in red) of fresh-frozen temporal arteries from patients with GCA or controls (first panel). Nuclei are stained with DAPI (blue). Co-expression (orange/yellow) is pointed with arrows and insets show magnified double-positive cells (scale bars in figures measure 100 μm and 15 μm for insets). (C) Summary panel of GM-CSF and GM-CSFRα expression by different cell types in three GCA-involved temporal arteries detected by immunofluorescence as in A and B. +++: 50%–100% positive cells; ++: 20%–40% positive cells; +: less than 20% positive cells; +/−: scattered cells; −: negative. DAPI, 4′,6-diamidino-2-phenylindole; GCA, giant cell arteritis; GM-CSF, granulocyte-macrophage colony stimulating factor; GM-CSFRα, GM-CSF receptor alpha chain; SMA, smooth muscle actin; TAB, temporal artery biopsy; VSMC, vascular smooth muscle cells.

Cell subsets potentially expressing GM-CSF and GM-CSFRα in GCA lesions were explored. As illustrated by immunofluorescence in figure 2, GM-CSF was mainly observed in macrophages and luminal endothelial cells and, to a lesser extent, in T cells, intimal myofibroblasts, and endothelial cells from vasa vasorum and neovessels. GM-CSFRα was detected mainly in macrophages, giant cells, endothelial cells and intimal myofibroblasts.

Serum GM-CSF concentration at diagnosis was 0.061±0.02 pg/mL (average±SEM) in patients with GCA and 0.035±0.02 pg/mL in controls (p=0.889).

### GM-CSF receptor-driven signalling pathways are activated in GCA lesions, and expression of molecules regulated by this pathway is increased

**Figure 3:**
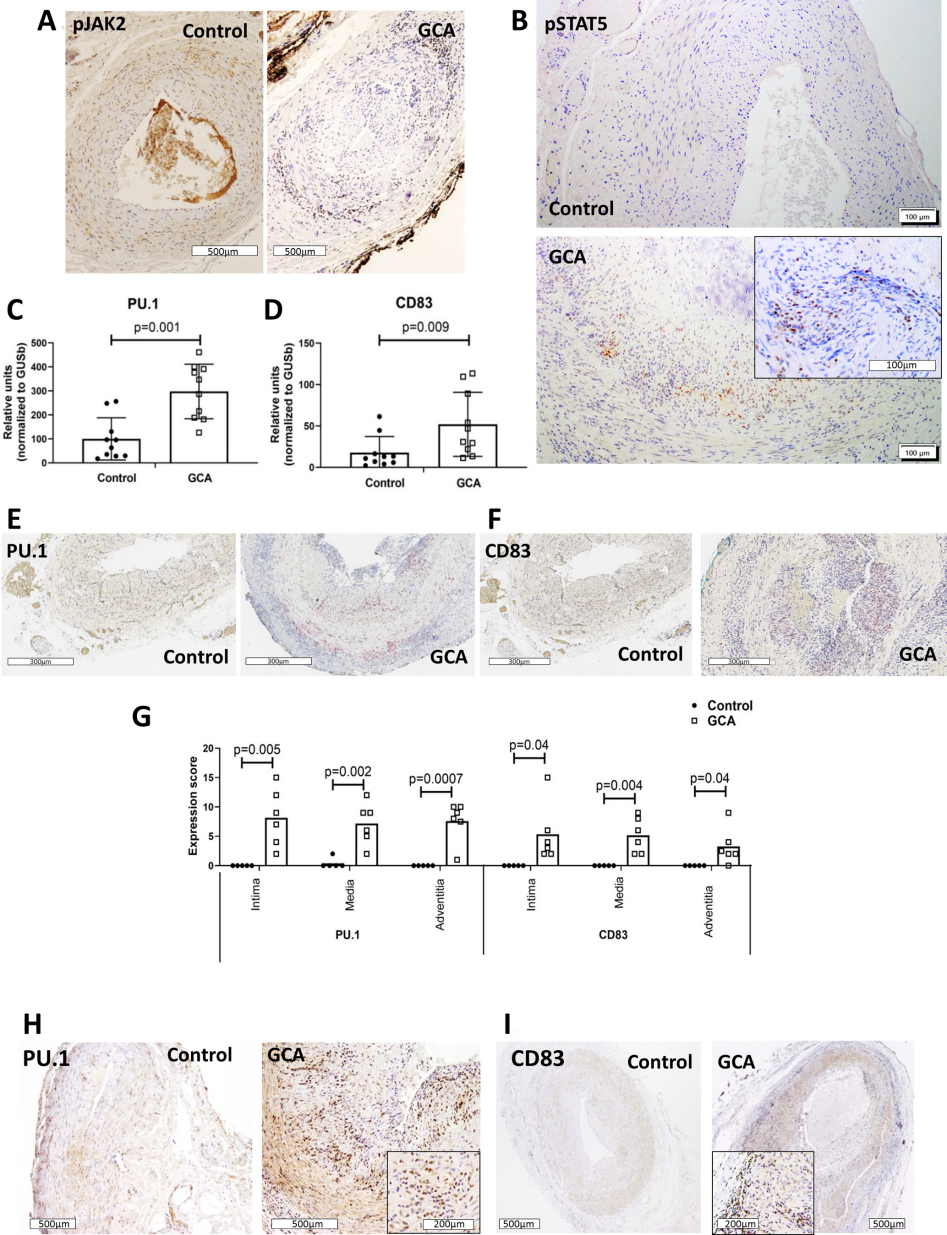
Activation of GM-CSFR-driven signalling pathways and target gene expression in GCA lesions. Immunostaining of histologically negative temporal artery biopsies (control) and GCA-involved arteries with anti-phospho-JAK2 (A) or anti-phospho-STAT5 (B) antibody (brown colour). Representative of 12 GCA and 5 control arteries. mRNA concentrations of PU.1 (C) and CD83 (D), in fresh-frozen control and GCA arteries (n=10 each group). PU.1 (E) and CD83 (F) RS images with positive red staining on control (n=5) and GCA temporal arteries (n=6), with their corresponding quantitation (G) in the intima, media and adventitia layers of the artery wall. Immunohistochemistry with anti-PU.1 (H) and anti-CD83 (I) antibodies on FFPE control and GCA arteries (brown). Representative of 12 GCA arteries and 5 controls. Magnification of each figure is indicated individually. FFPE, formalin-fixed paraffin-embedded; GCA, giant cell arteritis; GM-CSF, granulocyte-macrophage colony stimulating factor; GM-CSFRα, GM-CSF receptor alpha chain; RS, RNAScope.

After observing higher expression of GM-CSF and GM-CSFRα in GCA-involved arteries, signalling molecules downstream of GM-CSFR were examined. As shown in figure 3A, B and online supplemental figure S1, JAK2 and STAT5A, the main signalling proteins activated by GM-CSFR engagement, were phosphorylated in GCA lesions, and transcripts regulated by STAT5, such as Spi1 (PU.1) and CD83, were significantly increased in GCA arteries (figure 3C–G). CD83 and PU.1 protein, absent in controls, were clearly expressed in GCA arteries (figure 3H, I). PU.1 was detected in the nuclei, consistent with its function as transcription factor and suggestive of nuclear translocation on activation of upstream signalling. CD83 staining was more diffuse, possibly due to detection of its soluble form in addition to the membrane molecule.

10.1136/annrheumdis-2021-220873.supp2 Supplementary data

### GM-CSFRα inhibiting monoclonal antibody mavrilimumab reduces lymphocyte and myeloid cell markers in ex vivo cultured arteries from patients with GCA

**Figure 4:**
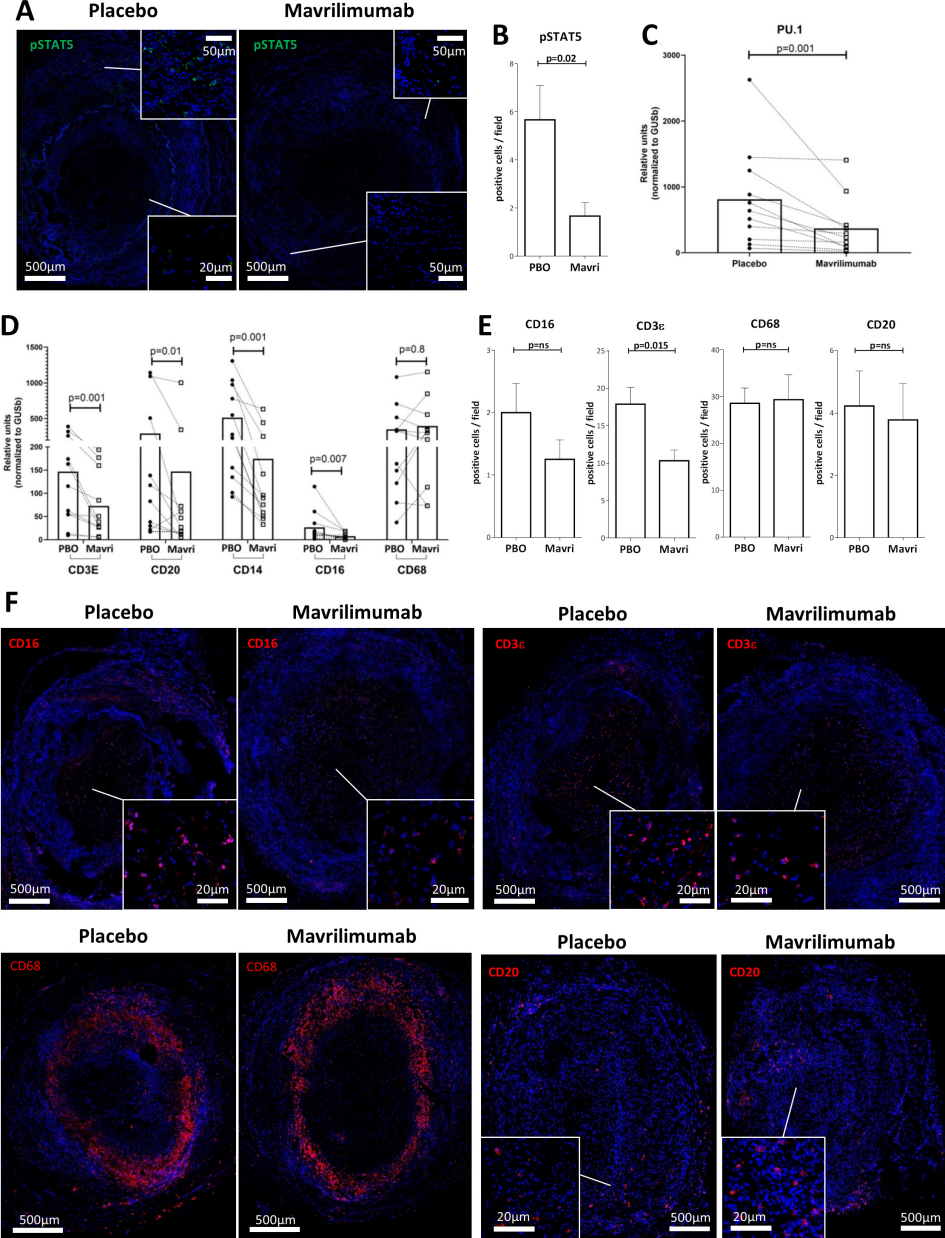
Effect of mavrilimumab on inflammatory infiltrates in ex vivo cultured arteries from patients with GCA. (A) Immunofluorescence staining with anti-phospho-STAT5 antibody (green) of a GCA artery cultured with placebo or mavrilimumab. (B) Quantification of positive cells per field A; this experiment was performed three times with similar results. (C) mRNA Spl1/PU.1 transcripts in 11 cultured GCA-affected temporal arteries in the presence of placebo or mavrilimumab. (D) Transcript levels for cell markers CD3ε, CD20, CD14, CD16 and CD68 in 11 cultured GCA-involved temporal arteries exposed to placebo or mavrilimumab. (E) Quantification of cells per field that are positive for anti-CD16, anti-CD3Ɛ, anti-CD68, and anti-CD20. (F) Immunofluorescence staining of cultured GCA-involved arteries in the presence of placebo or mavrilimumab with anti-CD16, anti-CD3Ɛ, anti-CD68, and anti-CD20 (red colour) and DAPI (blue). Representative of 3 GCA cultured arteries. Panel E is the quantification of panel F. DAPI, 4′,6-diamidino-2-phenylindole; GCA, giant cell arteritis.

To determine the contribution of GM-CSF to the above results and to assess the effects of GM-CSF pathway blockade on vascular inflammation, GCA arteries were cultured with anti-GM-CSFRα, mavrilimumab, for 5 days. Compared with placebo, treatment with mavrilimumab resulted in reduced phospho-STAT5 in lesions (figure 4A, B) and in lower mRNA expression of Spi1 (PU.1), a transcription factor that, along with STAT5, mediates GM-CSF effects (figure 4C).^28–30^ Furthermore, treatment with mavrilimumab resulted in significantly lower mRNA levels for T cell marker CD3ε, B cell marker CD20, monocyte marker CD14 and myeloid cell marker CD16 mRNAs (figure 4D). By contrast, no consistent changes were observed with transcripts for the macrophage marker CD68. Accordingly, fewer CD16 + and CD3ε + infiltrating cells and no change in CD68 + cells were observed by immunofluorescence (figure 4F). The reduction in CD20 transcripts, however, did not result from decreased numbers of B cells in tissue during the duration of mavrilimumab exposure (figure 4E, F).

### Mavrilimumab reduces expression of molecules involved in T cell activation and related to the Th1 differentiation pathway in ex vivo cultured arteries from patients with GCA

**Figure 5:**
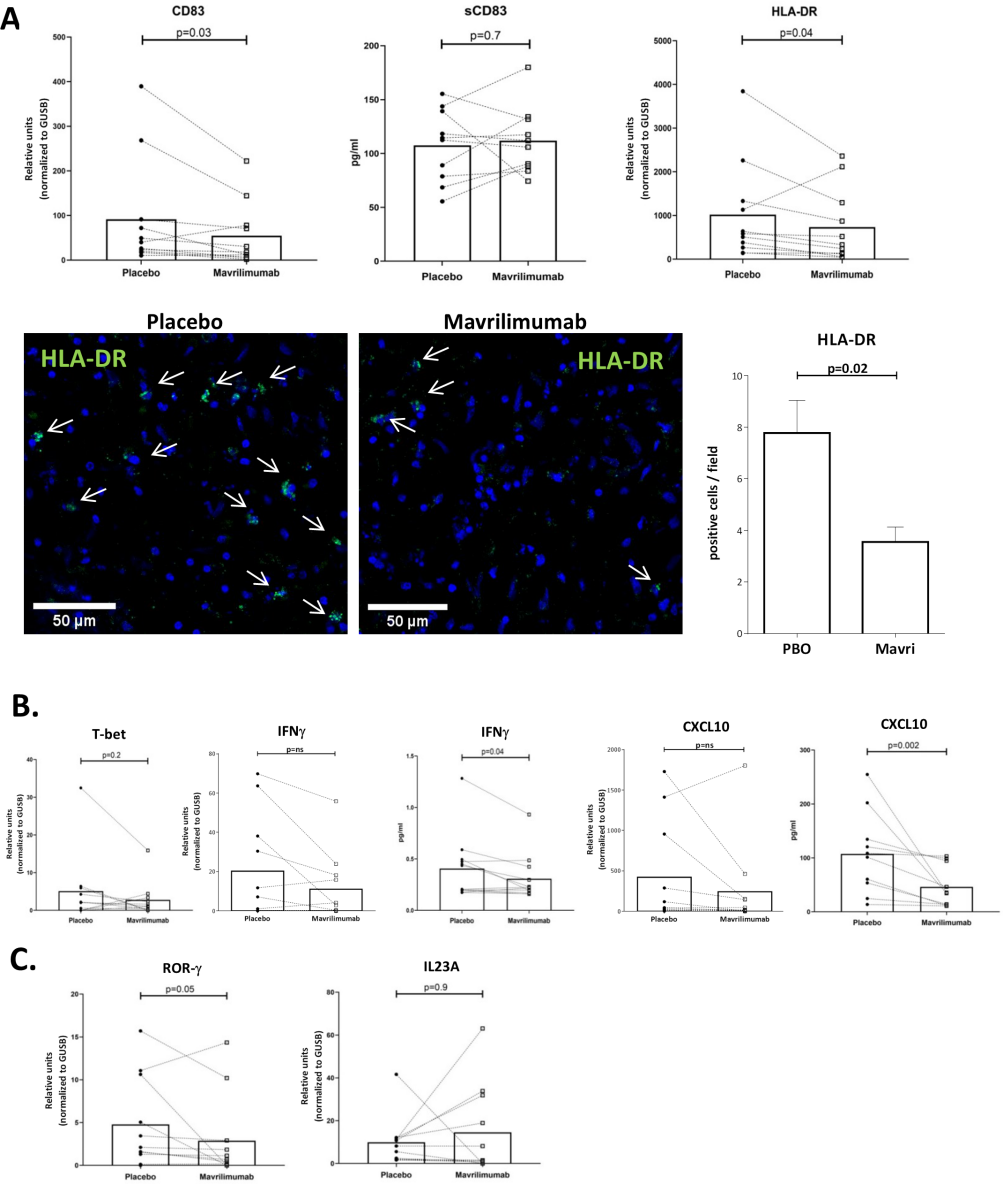
Mavrilimumab decreases molecules related to T lymphocyte activation and differentiation. (A) mRNA transcripts of CD83 (left) and HLA-DR (right) expressed in relative units and normalised to housekeeping gene *GUSB* in GCA-positive temporal arteries (n=11) cultured with placebo or mavrilimumab. Soluble CD83 measured (pg/mL) in supernatants of nine GCA cultured arteries exposed to placebo or mavrilimumab (central panel). Image shows HLA-DR expression by immunofluorescence in a GCA artery cultured with placebo or mavrilimumab. Images show detailed zoom amplification by confocal microscope with arrows indicating green HLA-DR-positive cells. Nuclei are stained with DAPI (blue). The graph on the right show the number of HLA-DR-positive cells per field in 9 fields per section. Immunofluorescence was performed in two GCA cultured arteries, with consistent results. (B) mRNA transcripts of *TBX21* (T-bet), *IFNG* (IFNγ) and CXCL10 in GCA arteries cultured with placebo or mavrilimumab (n=11). IFN-γ and CXCL-10 proteins were also measured in artery culture supernatants of the same specimens. Results are expressed in pg/mL. (C) *RORC* (ROR-γ) and *IL-23A* mRNA measurement in cultured GCA arteries treated with placebo or mavrilimumab. DAPI, 4′,6-diamidino-2-phenylindole; GCA, giant cell arteritis; HLA-DR, human leukocyte antigen-DR; IFN, interferon.

To further delineate the effects of mavrilimumab, expression of human leukocyte antigen-DR (HLA-DR) and CD83, relevant molecules to antigen presentation and T cell activation, was examined. Mavrilimumab significantly reduced HLA-DR and CD83 transcripts (figure 5A). Interestingly, concentration of the soluble, shed form of CD83, with counter-regulatory functions, did not decrease in the supernatant (figure 5A). HLA-DR reduction was also observed at the protein level (figure 5A).

To determine whether these effects resulted in decreased differentiation of T cells towards the Th1 or Th17 lineage, select markers were explored. Transcripts of master regulators of Th1 and Th17 differentiation, *TBX21* (T-bet) and *RORC* (RORγ), respectively, trended lower (figure 5B, C). Cytokines/chemokines related to Th1 differentiation pathway (interferon-γ (IFNγ) and CXCL10) trended lower (mRNA level) or were significantly lower (protein level) (figure 5B). IL-17A mRNA was virtually undetected in cultured arteries (data not shown), and IL-23p19 had disparate response among donors (figure 5C).

### Mavrilimumab decreases pro-inflammatory cytokines in ex vivo cultured arteries from patients with GCA

**Figure 6:**
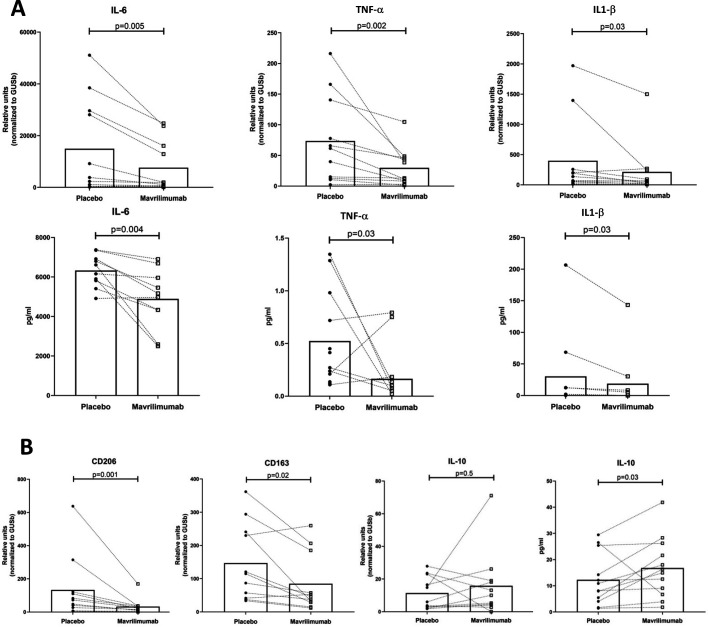
Mavrilimumab impacts macrophage functions. (A) Transcript levels of IL-6 (left), TNFα (central) and IL-1β (right) in GCA-positive arteries (n=11) exposed to placebo or mavrilimumab (mRNA, relative units). IL-6, tumour necrosis factor α (TNFα) and IL-1β proteins (pg/mL) were also measured in GCA artery culture supernatants of the same samples. (B) CD206, CD163 and IL-10 mRNA transcript levels in the same GCA arteries exposed to mavrilimumab or placebo. IL-10 protein (pg/mL) was also detected in the supernatant (right panel). GCA, giant cell arteritis; IL, interleukin.

Mavrilimumab elicited a significant reduction in the production and release of pro-inflammatory cytokines IL-6, TNFα and IL-1β, mostly but not exclusively produced by macrophages (figure 6A). Mavrilimumab also decreased markers of M2-like phenotype, including the mannose receptor CD206 and the scavenger receptor CD163 (figure 6B). A trend towards an increase in the anti-inflammatory cytokine IL-10 (mRNA and protein) was also observed (figure 6B).

Further supporting these results, recombinant human GM-CSF increased expression of the main transcripts decreased by mavrilimumab (online supplemental figure S2)

10.1136/annrheumdis-2021-220873.supp3 Supplementary data

### Mavrilimumab decreases mediators of vascular injury in ex vivo cultured arteries from patients with GCA

**Figure 7:**
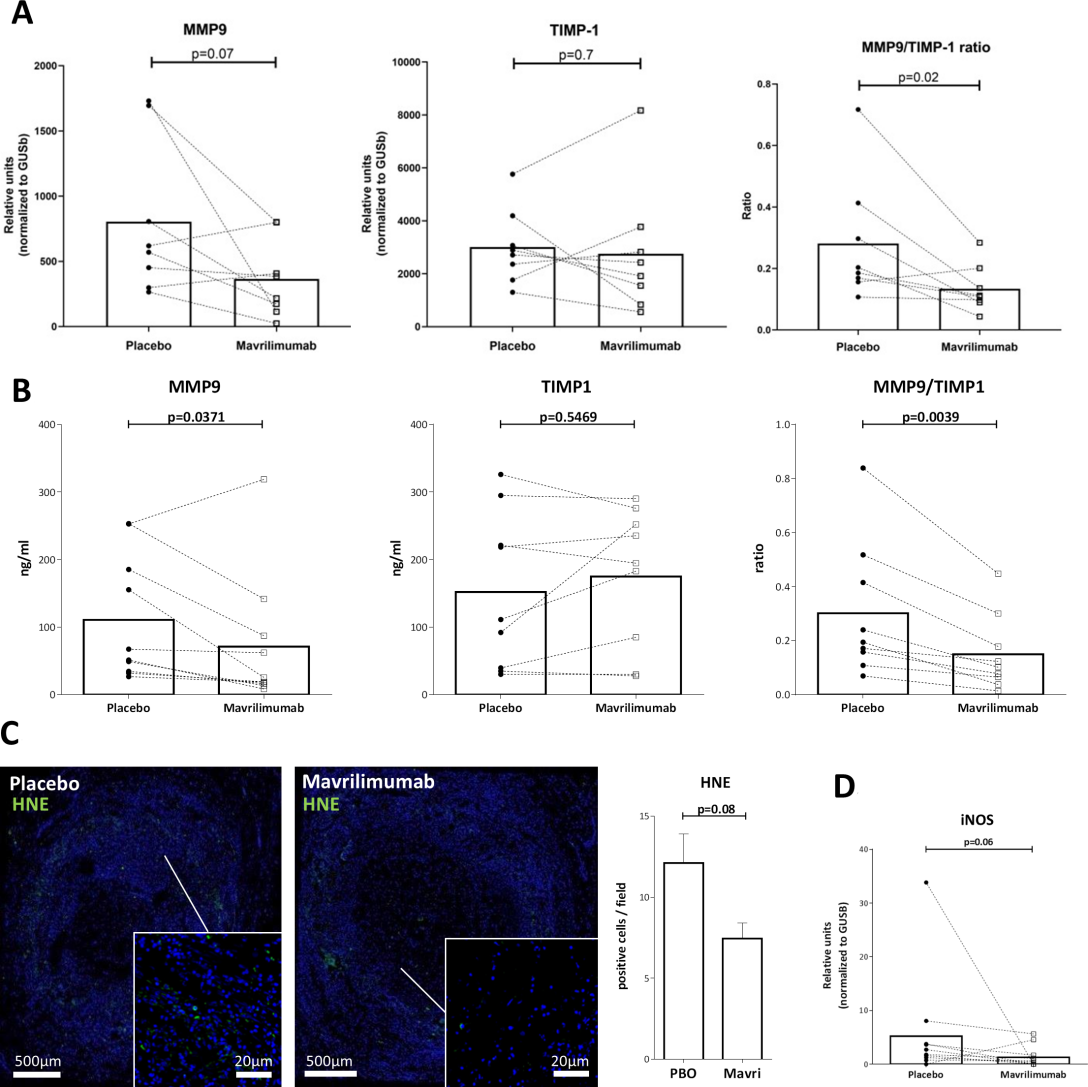
Effect of mavrilimumab on molecules related to vascular injury. (A) Transcripts of MMP-9, tissue inhibitor of metalloproteinases 1 (TIMP-1) and MMP-9/TIMP-1 mRNA ratio in 8 GCA-positive temporal arteries cultured with placebo or mavrilimumab. (B) MMP-9, TIMP-1 protein concentration and MMP-9/TIMP-1 protein ratio in the corresponding supernatants (ng/mL). (C) Immunofluorescence staining of HNE (green) with nuclei (in blue) in a GCA-involved artery cultured with placebo or mavrilimumab, and its quantitation (right panel). Immunofluorescence was performed in two GCA cultured arteries, with consistent results. (D) *NOS2* transcripts in 11 cultured GCA arteries exposed to placebo or mavrilimumab. GCA, giant cell arteritis; HNE, 4-hydroxynonenal; MMP-9, matrix metalloprotease 9.

Mavrilimumab decreased transcript and protein concentrations of the elastinolytic matrix metalloprotease 9 (MMP-9), whereas mRNA and protein of its natural inhibitor tissue inhibitor of metalloproteinases 1 (TIMP-1) remained unchanged, resulting in a significant decrease in proteolytic MMP-9/TIMP-1 balance (figure 7A, B). Mavrilimumab also reduced oxidative damage, as demonstrated by decreased presence of lipid peroxidation products (4-hydroxynonenal (HNE) protein adducts) in cultured arteries exposed to mavrilimumab as compared with placebo (figure 7C). *NOS2* (inducible nitric oxide synthase [iNOS]) mRNA expression also trended lower (figure 7D).

### Mavrilimumab reduces tissue angiogenesis in ex vivo cultured arteries from patients with GCA

**Figure 8:**
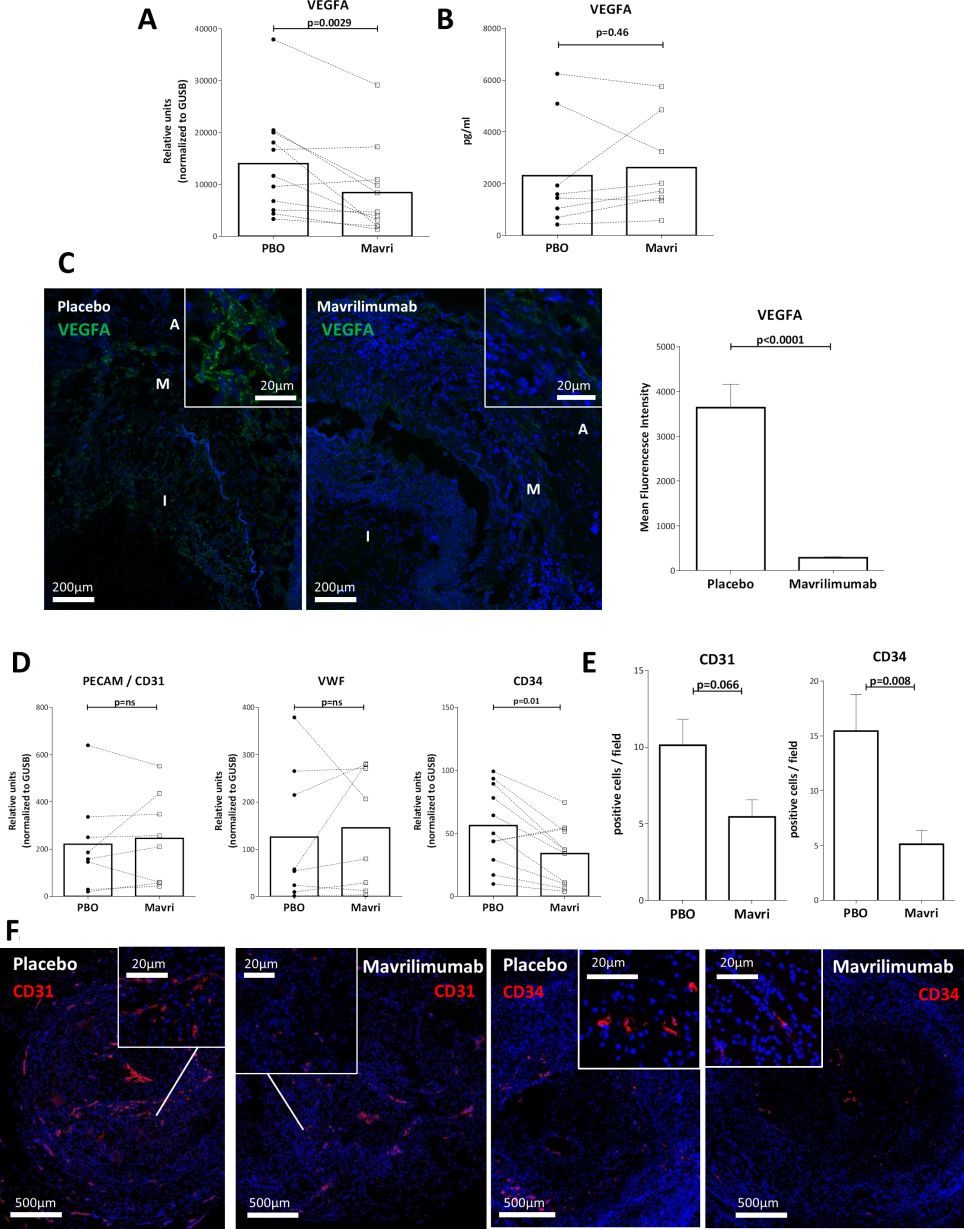
Mavrilimumab effect on angiogenesis. (A) Detection of vascular endothelial growth factor A (VEGFA) transcripts in 11 GCA-positive temporal arteries cultured with placebo or mavrilimumab. (B) Detection of VEGFA protein (pg/mL) in supernatants of eight respective arteries cultured with placebo or mavrilimumab. (C) Immunofluorescence with anti-VEGFA antibody of a GCA artery cultured with placebo or mavrilimumab (I, intima; M, media; A, adventitia). The graph on the right shows quantification of mean fluorescence intensity of the entire artery wall. (D) Measurement of PECAM-1 (n=8), vWF (n=8) and CD34 (n=11) transcripts in GCA cultured temporal arteries treated with placebo or mavrilimumab (relative units, normalised to housekeeping *GUSB*). (E) Quantification (positive cells per field) of immunofluorescence. Immunofluorescence was performed on two cultured biopsies with consistent results. (F) Immunofluorescence with anti-CD31 or anti-CD34 antibody of a GCA artery cultured with placebo or mavrilimumab. Inset images show zoom amplifications of positive (red) cells in areas of interest across the neointimal layer. Panel E is the quantification of panel F. GCA, giant cell arteritis.

Mavrilimumab reduced vascular endothelial growth factor A (VEGFA) mRNA in cultured arteries and VEGFA protein expression in tissue by immunofluorescence (figure 8A–C). However, no changes in VEGFA protein in the supernatant was observed, possibly due to its matrix-binding capacity and its autocrine/paracrine function.^37^ Based on the reduction of this important angiogenic factor, we explored the effects of mavrilimumab on endothelial cell markers and angiogenesis. Mavrilimumab did not elicit changes in constitutive endothelial cell marker vWF or CD31 mRNAs but a decrease in CD34 mRNA, expressed by neovessels and haematopoietic stem cells (HSC) was observed (figure 8D).^38 39^ Immunofluorescence showed a reduction in CD31 + and CD34+ neovessels within inflammatory lesions on exposure to mavrilimumab (figure 8E, F). Scattered CD34 + cells not aligned around a lumen were also observed in lesions and were reduced by mavrilimumab.

## Discussion

This study demonstrates expression of GM-CSFRα, the target of mavrilimumab, within the lesions of GCA-affected arteries and confirms the increased production of GM-CSF previously reported.^24 40 41^ Macrophages were the main cell type immunostained for GM-CSF and GM-CSFRα in inflamed arteries. Luminal endothelial cells and, to a lesser extent, intimal myofibroblasts and endothelial cells from vasa vasorum and neovessels also expressed GM-CSF along with a small subset of T cells, presumably ThGM-CSF cells.^30^ GM-CSFRα was expressed mainly by macrophages, endothelial cells and intimal myofibroblasts, suggesting that these cell types would be the most responsive to GM-CSF.

Contrary to a report in granulomatosis with polyangiitis,^42^ but similar to findings in other inflammatory conditions,^28–30^ GM-CSF was barely detectable in serum from patients with GCA, with no differences from healthy individuals. This supports a paracrine function of GM-CSF in the inflammatory microenvironment and limits the utility of serum GM-CSF as a biomarker of disease activity.

Detection of JAK2 and STAT5A phosphorylation in GCA lesions, along with increased expression of paradigmatic STAT5-regulated molecules, such as CD83 and transcription factor Spi1/PU.1,^43^ suggested activation of GM-CSF receptor-driven signalling pathways. Increased expression of additional relevant STAT5 or PU.1 regulated molecules, including major histocompatibility complex (MHC) class II molecule HLA-DR, adhesion molecules intercellular adhesion molecule 1 (ICAM-1) or vascular cell adhesion molecule 1 (VCAM-1), macrophage marker CD163, pro-inflammatory cytokines, such as IL-1 and TNFα, and metalloproteases such as MMP-9, has been previously demonstrated in GCA.^44–48^ Although these pathways can be activated by other cytokines, these data suggest active GM-CSF signalling in GCA arteries and a contribution of GM-CSF to the increased expression of key molecules involved in the pathogenesis of GCA.

To confirm the participation of GM-CSFR-mediated signalling in the increased expression of these and additional relevant molecules and inflammatory cell markers, cultured temporal arteries from patients with histopathologically proven GCA were exposed to mavrilimumab. Treatment with mavrilimumab resulted in significantly decreased transcripts of lymphoid markers, including B lymphocyte surface molecule CD20 and T lymphocyte surface glycoprotein CD3ε. A significant decrease in classical monocyte marker CD14 and myeloid cell marker CD16 mRNAs was also observed. In contrast, there was no consistent change in the expression of CD68, a scavenger receptor widely expressed by macrophages.

Mavrilimumab decreased expression of molecules produced by dendritic cells and B cells, which are essential for antigen-presenting function/T cell activation, such as CD83 and HLA-DR.^49 50^ This likely resulted in decreased Th1 differentiation, as indicated by reduced expression of Th1-related molecules, including IFNγ, TNFα and IFNγ-induced molecules such as CXCL10. Molecules related to Th17 differentiation, IL-1β and IL-6 were also decreased, but a more direct impact on IL-17 production could not be assessed. Although we and others have previously shown increased IL-17 expression in affected temporal arteries from patients with GCA,^18 21–23^ baseline expression of IL-17 was very low in cultured arteries, possibly related to previous GC treatment in the majority of patients^18^ or to the possible impact of culture on certain molecules.^36^

Mavrilimumab had a significant impact on pro-inflammatory functions of macrophages and endothelial cells, including expression of IL-1β, TNFα and IL-6, and expression of adhesion molecules for leucocytes. It also tended to increase expression and release of the anti-inflammatory cytokine IL-10, produced by regulatory T cells and B cells and M2-type macrophages.^51^ Mavrilimumab reduced MMP-9 expression with no change in expression of its natural inhibitor TIMP-1, thereby suggesting a shift in the MMP-9 proteolytic balance.^47^ Proteolytic enzyme MMP-9 has elastinolytic activity and may contribute to elastin degradation since it is expressed and activated in GCA lesions and in aortic tissue.^52^ MMP-9 may also contribute to GM-CSF-induced aneurysm formation, shown in an animal model.^53^ Macrophages present in GCA lesions have oxidative capacity as indicated by the presence of lipid peroxidation products (HNE) in GCA lesions.^27^ Treatment with mavrilimumab decreased HNE presence in cultured arteries indicating that mavrilimumab decreases oxidative damage in inflamed arteries.

The tuning in macrophage function induced by mavrilimumab does not parallel classical M1 (pro-inflammatory) or M2 (anti-inflammatory, reparative) phenotypes. Mavrilimumab reduced M1 markers, including HLA-DR and iNOS, and tended to increase M2 cytokine IL-10. However, mavrilimumab also reduced CD206 and CD163, which have been considered markers of M2 phenotype.^54^ It is important to remark that this distinction has been established mostly in in vitro differentiated macrophages or in murine models. In humans, plasticity of macrophages is far more complex.^54^ For example, macrophages co-expressing CD206 and MMP-9 have been observed in GCA lesions^41^ and a population of pro-inflammatory CD14+ HLA-DR^high^ CD206^+^ macrophages has been identified in human viral hepatitis.^55^ Overall, mavrilimumab decreased the inflammatory and destructive potential of macrophages.

GM-CSF influences endothelial cell behaviour and stimulates angiogenesis in experimental systems.^56^ Accordingly, mavrilimumab reduced microvessel density in GCA lesions. In addition to its potential direct effects, our results indicate that GM-CSF regulates VEGFA production. Since CD34 is expressed not only by endothelial cells from neovessels but also by HSC, which have recently been identified in chronic inflammatory lesions and promoted by GM-CSF,^57^ ^58^ we cannot exclude the possibility that some detected CD34 + cells were ectopic HSC. Mavrilimumab reduction of ectopic HSC may be a potential new relevant effect of mavrilimumab. Since neoangiogenesis is prominent in GCA lesions, and newly formed capillaries express adhesion molecules and recruit inflammatory leucocytes into arteries,^45 57^ mavrilimumab could indirectly reduce leucocyte recruitment by decreasing neoangiogenesis in addition to its direct effects on myeloid and other cells

Our study has limitations, including the relatively small number of cases investigated, inherent to the low incidence of GCA and the need of viable fresh tissue. In addition, our model explores changes induced by mavrilimumab in a target organ isolated from a functional immune system. However, the effects of mavrilimumab observed were consistent with the known functions of GM-CSF obtained in a variety of experimental systems. Furthermore, due to the small amount of available tissue, our experiments were limited to a single time-point. We cannot exclude that effects could be more prominent at other time points. Finally, most arteries were obtained from patients who had previously received GC treatment, as currently advised by international guidelines on GCA suspicion.^59^ Previous GC exposure reduces baseline expression of a variety of molecules, including GM-CSF.^36 60^ It would be possible that using treatment-naïve samples, changes would have been more prominent. However, this setting better reflects the real world and mavrilimumab still adds to potential GC effects on key inflammatory molecules.

In summary, this study reveals for the first time, functional changes induced by mavrilimumab in a classical target tissue of GCA. Mavrilimumab impacts inflammatory pathways considered relevant to the pathogenesis of vascular inflammation and injury, and the results from a recent phase 2 trial in which mavrilimumab was superior to placebo (both with 26-week prednisone taper) in reducing the risk of GCA flare and maintaining sustained remission^35^ validated the role of GM-CSF in GCA.

10.1136/annrheumdis-2021-220873.supp4 Supplementary video Video Abstract

## Data Availability

Data are available upon reasonable request. The individual anonymised data supporting the analyses contained in the manuscript will be made available upon reasonable written request from researchers whose proposed use of the data for a specific purpose has been approved. Data will not be provided to requesters with potential or actual conflicts of interest, including individuals requesting access for commercial, competitive or legal purposes. Data access may be precluded for studies in which clinical data were collected subject to legal, contractual or consent provisions that prohibit transfer to third parties. All those receiving access to data will be required to enter into a Data Use Agreement, which shall contain terms and conditions that are customary for similar agreements and similar companies in the industry. For requests, please email JFP, Kiniksa Pharmaceuticals's Chief Medical Officer, at jpaolini@kiniksa.com.
