# Supplementary material for: Blocking GM-CSF receptor α with mavrilimumab reduces infiltrating cells, pro-inflammatory markers and neoangiogenesis in ex vivo cultured arteries from patients with giant cell arteritis
Source: Ann Rheum Dis. 2022 Jan 19;81(4):524–36. doi: 10.1136/annrheumdis-2021-220873 (PMC8921590; doi:10.1136/annrheumdis-2021-220873)
Supplement: Supplementary data [file annrheumdis-2021-220873supp001.pdf]

SUPPLEMENTARY MATERIALS FOR:

BLOCKING GM-CSF RECEPTORα WITH MAVRILIMUMAB REDUCES INFILTRATING CELLS, PRO-INFLAMMATORY MARKERS, AND NEOANGIOGENESIS IN *EX-VIVO* CULTURED ARTERIES FROM PATIENTS WITH GIANT-CELL ARTERITIS

Marc Corbera-Bellalta[1]*, Roser Alba-Rovira[1*,] Sujatha Muralidharan[2]*, Georgina Espígol-Frigolé[1], Roberto Ríos-Garcés[1], Javier Marco-Hernández[1], Amanda Denuc[3], Farah Kamberovic[1], Patricia Pérez-Galán[4], Alexandra Joseph[2], Annalisa D'Andrea[2], Kent Bondensgaard[2], Maria C Cid[1]**, John F Paolini[2]**

* Equal contribution

** Shared senior authorship

[1]Vascullitis Research Group. Department of Autoimmune Diseases. Hospital Clínic. University of Barcelona, Institut d'Investigacions Biomèdiques August Pi I Sunyer (IDIBAPS). Barcelona. Spain

[2]Kiniksa Pharmaceuticals Corp., Lexington, Massachusetts

[3] Biobank. IDIBAPS. Barcelona. Spain

[4]Department of Hematology-Oncology, IDIBAPS, Barcelona, Spain

Supplemental material BMJ Publishing Group Limited (BMJ) disclaims all liability and responsibility arising from any reliance placed on this supplemental material which has been supplied by the author(s) *Ann Rheum Dis*

SUPPLEMENTARY METHODS

Patients

The study was performed with samples from 4 different groups of patients with suspected giant-cell arteritis (GCA). Group 1 consisted of 33 patients who donated the remnant of their diagnostic temporal artery biopsy (TAB) for research purposes as part of an approved and registered collection of frozen tissue (Vasculitis collection C.0003912). Sixteen biopsies were positive (with characteristic histopathological features of GCA) and 17 were negative and served as controls. Ten positive and 10 negative biopsies, randomly selected among those from patients who had not received glucocorticoid treatment at the time of the temporal artery biopsy, were processed for RNA extraction. The remaining 12 (6 positive and 7 negative) were used for immunofluorescence or Western-blot studies. These patients had received glucocorticoid treatment for ≤ 3 days. Group 2 consisted of 23 anonymous donors subjected to TAB for suspected GCA. Eighteen were positive and 5 negative for GCA diagnosis. Samples were formaldehyde fixed and paraffin embedded (FFPE) and were used for in situ RNA hybridization and immunohistochemical detection of the molecules of interest. These samples were purchased from Tissue for Research biobank but clinical data from their donors were limited. Group 3 consisted of 60 patients and 12 healthy donors of similar age and sex distribution who donated serum for the above-mentioned collection. Group 4 consisted of 16 patients diagnosed with biopsy-proven GCA during the study period (October 2018-August 2021) and cultured *ex-vivo* for the experiments described below. Clinical characteristics of patients and controls are depicted in Supplementary Table S1 and are representative of published GCA series.

Control patients who donated their biopsies for the collection, had the following final diagnosis: non-arteritic ischemic optic neuropathy (5 patients), non-specific constitutional symptoms in pluripatologic patients (7 patients), and non-specific headache (5 patients). In all of them the clinical suspicion was low and biopsies were performed to further rule-out GCA. None of them received prolonged glucocorticoid therapy.

Temporal artery culture

Serial, 1 mm thick sections of fresh temporal artery fragments from 16 patients with GCA were cultured *ex-vivo* on ice-cold reconstituted basement membrane *Matrigel*^{TM} (BD Biosciences, San Jose, California, USA), as previously described (Corbera-Bellalta, M. et al, ARD 2014). Each section was cultured in Roswell Park Memorial Institute-1640 (RPMI-1640) medium (Lonza, Verviers, Belgium) supplemented with 10% fetal bovine serum (FBS; Gibco, Life Technologies, Waltham, Massachusetts, USA), 2mM of L-glutamine (Gibco), 50µg/mL of gentamicin (Braun, Melsungen, Germany) and 2.5 µg/mL of amphotericin B (Invitrogen). TAB sections were exposed to mavrilimumab (20µg/mL, Kiniksa, Lexington, MA, USA), placebo (mavrilimumab formulation buffer) (Kiniksa) or rhGMCSF (20ng/ml, R&D Systems). Each condition was tested in 2–3 replicate wells. TAB sections were cultured for 5 days at 37°C and 5% CO_2. The supernatant fluid was centrifuged and stored at -80ºC until use. Cultured arteries were processed for RNA extraction (11 samples) or immunofluorescence (5 samples). Since the primary purpose of temporal artery biopsy is supporting diagnosis, the fragment spared for culture was small and limited number of conditions/markers per biopsy could be tested.

BMJ Publishing Group Limited (BMJ) disclaims all liability and responsibility arising from any reliance placed on this supplemental material which has been supplied by the author(s)

RNA extraction and reverse-transcription

Samples were homogenized in 1mL of TRI-Reagent (MRC Inc) prior to RNA extraction through chloroform-isopropanol separation-precipitation method. Pellets were rinsed with ethanol (70%) (Panreac Applichem, Barcelona, Spain). Total RNA was quantified by spectrophotometry (Quawell Technology, San Jose, CA, USA), and 1µg of RNA was reverse transcribed to cDNA employing High Capacity cDNA Reverse Transcription kit (Applied Biosystems, Foster City, California, USA) in a final volume of 100µL. Samples were stored at -80ºC until use.

Candidate gene expression analysis

Total RNA was extracted from cryostat sections of OCT-embedded frozen biopsies or cultured biopsies, homogenized with Bullet Blender (Next Advance, Troy, NY, USA) using TRI-Reagent (MRC Inc, Cincinnati, OH, USA).

cDNAs were obtained by reverse-transcription and measured by quantitative real-time PCR with specific pre-developed TaqMan probes (Applied Biosystems) (Supplementary Table S3).

Fluorescence was detected using ABI Prism 7900 Hardware Real-Time PCR System and results were quantified and analyzed with Sequence Detector software v.2.4 (Applied Biosystems). Gene expression was normalized to the expression of the endogenous control gene GUSB using comparative ΔCt method and expressed in relative units to GUSB expression.

Immunofluorescence of TABs

Fresh-frozen or cultured biopsies were fixed with 4% paraformaldehyde (PFA), pre-rinsed with increasing concentrations of sucrose, 15% and 30%, before being embedded in Tissue-Tek OCT Compound (Sakura, Flemingweg, The Netherlands) and preserved at -80°C until use. Sections of 7µm were obtained with a cryotome cryostat (Leica Microsystems), re-fixed with 4% PFA, permeabilized with 0.1% Triton solution and blocked with 5% donkey serum (Sigma) in 0.1% Triton PBS1X. Primary and secondary antibodies used and their concentrations or specific dilutions and corresponding sources are detailed in Supplementary table S4. Mounting medium with 4',6-diamidino-2-phenylindole (DAPI Fluoromount-G, Southern Biotech, Birmingham, AL, USA) was used to stain the nuclei and to preserve fluorescence. To control for non-specific background, for each condition, some sections were processed omitting the primary antibody. Immunofluorescence samples were observed with confocal microscopy SP5 Leica (Leica Microsystems) and LSM880 (ZEISS) and the images were analyzed using the ImageJ software (National Institutes of Health, Wayne Rasband, Bethesda, Maryland, USA).

Modified RIPA buffer composition

Commercial RIPA lysis buffer (Sigma-Aldrich, Ayrshire, UK) was supplemented with phenylmethylsulfonyl fluoride (PMSF) (1mM) (Sigma), benzenesulfonyl fluoride hydrochloride (1mM) (Roche), orthovanadate (2nM) (Sigma), leupeptin (Sigma), aprotinin (Thermo Scientific), and pepstatin (Sigma) (0,5ug/mL), ethylene-diamine-tetraacetic acid (EDTA) (1mM) (Sigma), ethylene-glicol-tetraacetic acid (EGTA) (1mM) (Sigma), sodium fluoride (NaF) (50mM) (Sigma), and NP-40 detergent (1%) (Abcam).

Western Blot

TABs were homogenized with Bullet Blender (Next Advance, Try, NY) in complete RIPA buffer supplemented with protease and phosphatase inhibitors. Protein lysates were resolved on sodium dodecyl sulfate (SDS)-polyacrylamide electrophoresis gels (BioRad) under reducing conditions and transferred to nitrocellulose membranes. Primary and secondary antibodies used with their specific dilutions and corresponding sources are detailed in Supplementary table S4. When necessary, membranes were treated with stripping buffer (100mM glycine (Sigma) and 100mM NaCl (Sigma); pH 2.5). Chemiluminescence signal was measured with the ImageQuant LAS-4000 imaging system (GE HealthCare Life Science, Pittsburgh, PA, USA). Images were analyzed and quantified using the ImageJ software (National Institutes of Health, Wayne Rasband, Bethesda, Maryland, USA).

Detection of proteins in the supernatants of cultured arteries and patient sera

Cytokines, chemokines, or membrane-bound molecules released into artery culture supernatants or present in serum were detected by immunoassay. Sources and characteristics of the commercially available immunoassays used are depicted in Supplementary Table S5. All procedures were performed according to the instructions of the respective manufacturers.

Supplementary Table S1: Clinical characteristics of GCA and control sample donors

	Fresh-frozen GCA arteries (n=16)*	Fresh-frozen control arteries (n=17)**	Formaldehyde fixed, paraffin embedded GCA arteries (n=12)†	GCA Serum (n=60)	Cultured GCA arteries (n=16)***
**Age, median (range) years**	80 (69-90)	78.5 (62-89)	75 (64-88)	77 (57-90)	80 (66-93)
**Sex**					
**Male, n (%)**	6 (37.5%)	7 (41%)	4 (33.3%)	21 (35%)	5 (31.25%)
**Female, n (%)**	10 (62.5%)	10 (59%)	8 (66.7%)	39 (65%)	11 (68.75%)
**Clinical data at diagnosis**					
**Cranial symptoms (%)**					
**Headache, n (%)**	12 (80%)	7 (46.7%)	8 (66.7%)	49 (81.7%)	14 (87.5%)
**Scalp tenderness, n (%)**	6 (40%)	3 (20%)	N/A	24 (40%)	8 (50%)
**Jaw claudication, n (%)**	7 (46.7%)	2 (13.3%)	N/A	25 (41.7%)	10 (62.5%)
**Stroke/visual events, n (%)**	4 (25%)	5 (33.3%)	4 (33.3%)	17 (28.3%)	5 (31.25%)
**Systemic symptoms, n (%)**					
**Fever, n (%)**	4 (26.7%)	1 (6.7%)	N/A	21 (35%)	3 (18.75%)
**Weight loss, n (%)**	7 (46.7%)	9 (60%)	N/A	23(38.3%)	5 (31.25%)
**Polymyalgia rheumatica, n (%)**	4 (26.7%)	3 (20%)	N/A	22(36.7%)	3 (18.75%)
**Laboratory findings at diagnosis**					
**ESR, mm/h**	81.5 ± 25	89 ± 39	N/A	90 ± 32	77 ± 47
**CRP, mg/dL**	5.20 ± 4.23	2.97 ± 4.05	N/A	9.41 ± 7.61	11.97 ± 11.3
**Haemoglobin, g/L**	108.5 ± 12	112 ± 19	N/A	112 ± 15	118.9 ± 19
**Prednisone treatment pre-biopsy or serum extraction, n (%)**	4 (25%)	5 (33.3%)	N/A	5 (8.5%)	12 (75%)

* 10 for RNA extraction, 3 for protein extraction (western-blot), and 4 for immunofluorescence.

**10 for RNA extraction, 3 for protein extraction (western-blot) and 4 for immunofluorescence.

*** 11 for RNA extraction, 5 for confocal microscopy and 13 for ELISA.

† Some relevant clinical information was not available from purchased samples.

Supplementary Table S2: Quantitation of RNAscope signal

Expression score was calculated as RS score (dots/cell) multiplied by Positivity score (% cells positive with >1 dot/cell).

RS score	Criteria
0	No staining or <1 dot/10 cells
1	1-3 dots/cell
2	4-9 dots/cell
3	10-15 dots/cell
4	>15 dots/cell

Positivity score	Criteria
1	<25% cells positive
2	25-50% cells positive
3	50-75% positive
4	>75% cells positive

Supplemental material BMJ Publishing Group Limited (BMJ) disclaims all liability and responsibility arising from any reliance placed on this supplemental material which has been supplied by the author(s) *Ann Rheum Dis*

Supplementary Table S3: Probes used for real-time quantitative RT-PCR

Gene name	Probe reference	Gene name	Probe reference
**GUSB**	Hs99999908_m1	**CD14**	Hs00169122_g1
**TBX21 (T-bet)**	Hs00894392_m1	**CD16**	Hs04334165_m1
**RORC (ROR-[illegible]O**	Hs01076112_m1	**CD3E**	Hs01062241_m1
**IL6**	Hs00985639_m1	**CD20**	Hs00544818_m1
**IL1B (IL-1β)**	Hs01555413_m1	**CD68**	Hs00154355_m1
**TNFa (TNFα)**	Hs00174128_m1	**CD83**	Hs00188486_m1
**IFNg (IFN-γ)**	Hs00174143_m1	**SPI1 (PU.1)**	Hs02786711_m1
**IL17A**	Hs00174383_m1	**HLA-DRA**	Hs00219575_m1
**IL23a**	Hs00372324_m1	**NOS2 (iNOS)**	Hs01075529_m1
**CXCL10**	Hs00171042_m1	**GM-CSF**	Hs00929873_m1
**IL10**	Hs00961622_m1	**GM-CSFRA (GM-CSFRα)**	Hs00538896_m1
**CD163**	Hs00174705_m1	**MMP-9**	Hs00234579_m1
**CD206**	Hs00267207_m1	**TIMP1**	Hs00171558_m1
**PECAM-1 (CD31)**	hS00169777_m1	**VWF**	Hs01109446_m1
**CD34**	Hs00990732_m1	**VEGF-A**	Hs00900055_m1

Supplementary table S4: List and characteristics of antibodies

A.

Primary antibodies used for immunofluorescence

Antibody	Company	Working concentration or dilution	Host animal	Clonality (clone/ref)
Anti-CD68	DAKO	undiluted	Mouse	Monoclonal (KP1)
Anti-CD3	Thermo Fisher	1:200	Mouse	Monoclonal (F7.2.38)
Anti CD3ε	Raybiotech	1μg/ml	Rabbit	Polyclonal (119-11933)
Anti-CD16	BioRad	1:40	Mouse	Monoclonal (2H7)
Anti-CD20	DAKO	undiluted	Mouse	Monoclonal (L26)
Anti-CD31	DAKO	undiluted	Mouse	Monoclonal (JC70A)
Anti-α-SMA	Abcam	4,5μg/ml	Mouse	Monoclonal (ab54723)
Anti-GMCSF	Abcam	5μg/mL	Rabbit	Polyclonal (ab220888)
Anti-GMCSFRα	Biorbyt	10μ/ml	Rabbit	Polyclonal (orb157207)
Anti-HLA-DRA	Beckton Dickinson	1:100	Mouse	Monoclonal (G46-6)
Anti-HNE	Bioss Antibodies	20μg/ml	Rabbit	Polyclonal (bs6313R)
Anti-pSTAT5 (Tyr694)	Cell Signaling	1:200	Rabbit	Polyclonal (C71E5)
Anti-CD34	Ventana	0,8μg/ml	Mouse	Monoclonal
Anti-VEGFA	Santa Cruz Biotechnology	4μg/ml	Rabbit	Polyclonal (sc-152)

Secondary antibodies used for immunofluorescence

Antibody	Company	Working concentration or dilution	Host animal	Clonality (clone/ref)
Anti-Mouse-Alexa Fluor 555	Molecular Probes	6,6μg/ml	Donkey	Polyclonal (A31570)
Anti-Rabbit-Alexa Fluor 488	Molecular Probes	6,6μg/ml	Donkey	Polyclonal (A21206)

B.

Primary antibodies used for Western-Blot

Antibody	Company	Working concentration or dilution	Host animal	Clonality (clone/ref)
Anti-pSTAT5 (Tyr694)	Cell Signaling	1:1000	Rabbit	Polyclonal (C71E5)
Anti-STAT5	Cell Signaling	1:1000	Rabbit	Polyclonal (D3N2B)
Anti-β-ACTIN	Sigma	1:5000	Mouse	Monoclonal (AC-15)

Secondary antibodies used for immunoblot

Antibody	Company	Working concentration or dilution	Host animal	Clonality (clone/ref)
Anti-Rabbit-HRP	Cell	1:2000	Goat	Polyclonal (7074S)
Anti-Mouse-HRP	BioRad	1:2000	Goat	Polyclonal (170-6516)

C.

Primary antibodies used for immunohistochemistry

Antibody	Company	Working concentration or dilution	Host animal	Incubation time	Clonality (clone/ref)
Anti-CD83	BioRad	100μg/ml	Mouse	1 hour	Monoclonal (HB15-E)
Anti-GMCSFRα	Biorbyt	10μg/ml	Rabbit	1 hour	Polyclonal (orb157207)
Anti-GMCSF	My Biosource	10μg/ml	Mouse	2 hours	Monoclonal (7U1)
Anti-pJAK2	Abcam	2,5μg/ml	Rabbit	1 hour	Polyclonal (ab32101)
Anti-PU.1	My Biosource	2,5μg/ml	Goat	1 hour	Polyclonal
Anti-pSTAT5	Cell Signaling	1:150	Rabbit	1 hour	Polyclonal (C71E5)

Note: Concentration of some antibodies was not detailed in the technical information, and the working dilution of the purchased material is provided

Supplementary Table S5: ELISA kits used for immunoassay

Target protein	Source	Sensitivity threshold	Samples used
GM-CSF	R&D Systems	> 0.26 pg/mL	Serum
IL-6	R&D Systems	> 0.7 pg/mL	Culture supernatants
TNFα	R&D Systems	0.011-0.049 pg/mL	Culture supernatants
IL-1β	R&D Systems	> 1 pg/mL	Culture supernatants
IFNγ	R&D Systems	0.025-0.173 pg/mL	Culture supernatants
CXCL10	R&D Systems	0.41-4.46 pg/mL	Culture supernatants
MMP-9	R&D Systems	0.002-0.01 ng/mL	Culture supernatants
IL-10	R&D Systems	>3.9 pg/mL	Culture supernatants
sCD83	Antibodies Online	> 1.42 pg/mL	Culture supernatants
TIMP-1	R&D Systems	> 0.08 ng/mL	Culture supernatants
VEGF-A	Invitrogen	> 7.9 pg/mL	Culture supernatants
