# Supplementary material for: Blocking GM-CSF receptor α with mavrilimumab reduces infiltrating cells, pro-inflammatory markers and neoangiogenesis in ex vivo cultured arteries from patients with giant cell arteritis
Source: Ann Rheum Dis. 2022 Jan 19;81(4):524–36. doi: 10.1136/annrheumdis-2021-220873 (PMC8921590; doi:10.1136/annrheumdis-2021-220873)
Supplement: Supplementary data [file annrheumdis-2021-220873supp002.pdf]

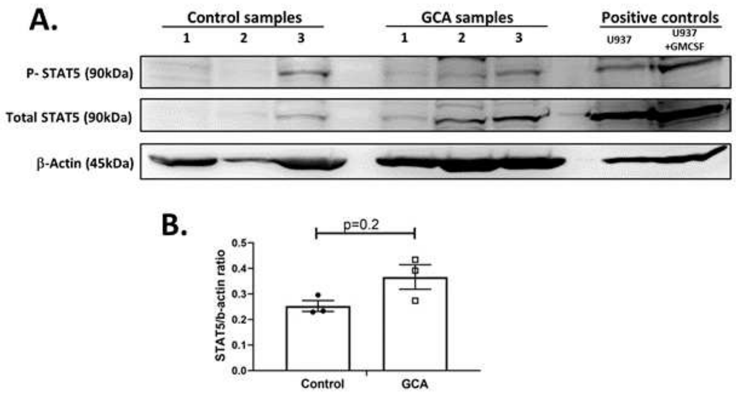

Supplementary Figure S1: **STAT5 activation in GCA lesions**

Immunoblot (A) and its corresponding densitometric quantitation (B) of total STAT5 or STAT5 phosphorylated at the tyrosine 694 residue, in lysates of fresh temporal arteries from 3 controls and 3 GCA patients, with β-actin expression as control for loading. Lysates of U937 cell line with or without exposure to recombinant GM-CSF at 20 ng/ml were used as a positive control for STAT5 expression and phosphorylation.
