# Supplementary material for: Blocking GM-CSF receptor α with mavrilimumab reduces infiltrating cells, pro-inflammatory markers and neoangiogenesis in ex vivo cultured arteries from patients with giant cell arteritis
Source: Ann Rheum Dis. 2022 Jan 19;81(4):524–36. doi: 10.1136/annrheumdis-2021-220873 (PMC8921590; doi:10.1136/annrheumdis-2021-220873)
Supplement: Supplementary data [file annrheumdis-2021-220873supp003.pdf]

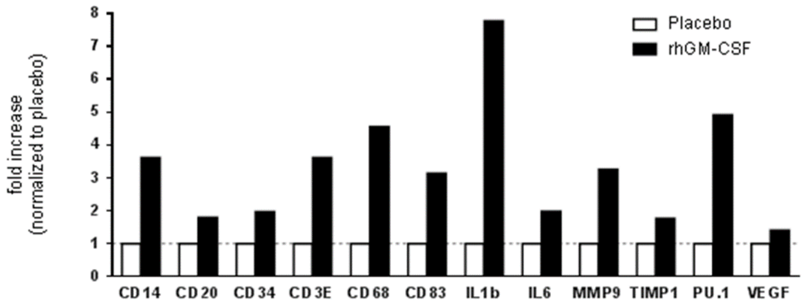

Supplementary Figure S2: **GM-CSF effect on GCA lesions**

Transcript measurement of relevant pro-inflammatory cytokines and cell markers from a GCA positive temporal artery biopsy cultured in presence of placebo or recombinant human GM-CSF (rhGM-CSF) at 20ng/ml, given as fold increase, normalized to placebo.
